# Mathematical modeling of a continuous-flow packed-bed reactor with immobilized lipase for kinetic resolution of (R,S)-2-pentanol

**DOI:** 10.3906/kim-1910-22

**Published:** 2020-10-26

**Authors:** Aslı SOYER MALYEMEZ, Abdulwahab GIWA, Emine BAYRAKTAR, Ülkü MEHMETOĞLU

**Affiliations:** 1 Department of Chemical Engineering, Faculty of Engineering, Ankara University, Ankara Turkey; 2 Chemical and Petroleum Engineering Department, College of Engineering, Afe Babalola University, Afe Babalola Way, Ado-Ekiti, Ekiti State Nigeria

**Keywords:** (R, S)-2-pentanol, anti-Alzheimer drug intermediate, mathematical modeling of a packed-bed reactor, axial dispersion, kinetic resolution

## Abstract

In this study, the kinetic resolution of (R,S)-2-pentanol via transesterification to achieve S-2-pentanol, a key intermediate required in the synthesis of anti-Alzheimer drugs, was investigated in continuous-flow packed-bed reactors. The effects of residence time, substrate concentration, and operation time of the enzyme were investigated. Under steady state conditions, 50% conversion and enantiomeric excess of the substrate (ee _S_ >99% were achieved at a residence time of 0.04 min. Productivity of the continuous-flow process (1.341 mmol/min/g)was about 4 times higher than that of the corresponding batch process (0.363 mmol/min/g). In addition, the mathematical modeling of the packed-bed reactor was conductedusing an axial dispersion model. Ping Pong Bi Bi kinetics was used in this model. Design parameters were determined and the developed equations were solved using an algorithm for solving boundary value problems for ordinary differential equations by collocation (bvp4c) using MATLAB. The results, obtained from the model, fitted the experimental data very well.)

## 1. Introduction

Optically pure secondary alcohols and their derivatives are widely used as building blocks in the chemical and pharmaceutical industries. For example, (S)-2-pentanol is used in anti-Alzheimer drug production. Lipases are frequently used in stereoselective biotransformation [1–5].Due to the different biological effects of enantiomers, it is important to use enantiomerically pure compounds in drugs. The kinetic resolution, which depends on the different reaction rates of the enantiomers, is a more economical and popular (common) method of producing optically active pure enantiomer from its racemic mixture (racemate).

The optical purity of the product (enantiomerically pure compound) depends on the enantioselectivity of the catalyst. Additionally, the reaction configuration is an important parameter from the point of high productivity [6]. Moreover, in order to obtain maximum enantioselectivity with kinetic resolution, the reaction must be terminated after a certain time. This can be performed via regulation of the flow rate; in other words, the residence time in the continuous-flow system. The production of (S)-2-pentanol, using immobilized lipase, with a continuous-flow packed-bed reactor has not been investigated in detail thus far.

In recent years, continuous-flow processes have become a more frequent method for the production of enantiomerically pure compounds [7]. Large-scale enzymatic resolution in a packed-bed reactor was first used in Japan in 1966 [8]. There are a few examples of hydrolase-catalyzed enantioselective processes conducted in continuous-flow systems.Kinetic resolution of (R,S)-1-phenylethanol was investigated in batch and continuous-flowpacked-bed reactors. It was reported that the performance of Chirazyme L2 was affected in a packed-bed reactor, due to mass transfer limitations and enzyme compaction [9].In another study, the kinetic resolution of 1-phenylethanol was performed in acontinuous-flow packed-bed reactor using immobilized *Candida antarctica* lipase B. It was found that the continuous-flow reactions were successfully performed for the kinetic resolution of 1-phenylethanolwithout enantioselectivity changes related to the reactor configuration, and the productivity (specific rate) of the lipases was higher in the continuous-flow reactor than in a batch reactor [2]. In a study of organo-catalystα-amination of aldehydes, excellent enantioselectivities (90%–99%ee) were obtained in a packed-bed reactor. Additionally, it was observed that no decrease in catalyst activity or selectivity was detected during the continuous-flow operation for a long period of time [10]. The enzymatic resolution of racemic 2-acetoxy-2(2’-chlorophenyl) acetate was investigated using free and immobilized enzymes. Higher productivity and enzyme stability were obtained with immobilized enzymes in continuous-flow operation than in a batch operation [11].

**Figure 1:**
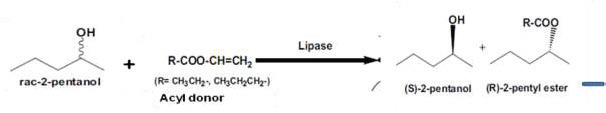
Transesterification reaction of (R,S)-2-pentanol with vinyl ester in hexane medium with lipase.

The modeling of reactors for industrial production is of great importance. In this study, kinetic resolution of (R,S)-2-pentanol (Figure 1) was studied in a continuous-flow packed-bed reactor. The effect of residence time (flow rate), substrate concentration, and enzyme operation time were investigated. In addition, the packed-bed reactor design model was investigated and equations of the proposed model were solved using MATLAB (MathWorks, Inc., Natick, MA, USA).

## 2. Materials and methods

### 2.1. Materials

Novozyme 435, lipase B from *Candida antarctica* immobilized on acrylic resin, was obtained from Novozymes A/S (Frederiksberg, Denmark). (R,S)-2-pentanol and vinyl butyrate were procured from Sigma-Aldrich Chemie GmbH (Taufkirchen, ) and Fluka (Tokyo, Japan), respectively. Ethyl propionate and *n* -hexane were obtained from Merck KGaA (Darmstadt, Germany). All of the chemicals were analytical grade and used without any pretreatment.

### 2.2. Operation ofthe reactor and transesterification reaction

The transesterification reaction of the (R,S)-2-pentanol was performed with a continuous-flowreactor, which had length-to-diameter (L/D)ratio of 12.5. Dimensions of this reactorwere 5-cm in length, 0.4-cm in internal diameter, and 0.628-mL in total volume. The stainless steel gas chromatograph guard column was filled with 270 mg of Novozyme 435 (with a particle diameter (dp) of 0.5 mm), so that the void fractionε)was 0.3. Before packing, the column was washed with ethanol and distilled water 5 times. The reaction temperature was constant at 30 °C, by means of a heat jacket with heater tape. Reaction mixture was pumped into the reactor up flow direction at different flow rates with a peristaltic pump (0.1–1.0 mL/min). In order to stabile the catalyst bed, the column was sealed with a silver metal filter membrane. Samples (400 mL) were taken from upstream at regular time intervals, diluted with ( *n* -hexane,cooled in ice bath to stop the reaction, and then kept in freezer –18°C) until analysis. The experiments were performed in duplicate.(

### 2.3. Analytical model

Analysis was performed on a Shimadzu GC-2010 gas chromatograph (Kyoto, Japan) equipped with a flame ionization detector (FID) andβ-DEX-120 chiral capillary column (Supelco, Sigma-Aldrich Chemie GmbH) with helium as the carrier gas. The injection and FID temperatures were set at 250 °C and the oven temperature was held at 50°C for 10 min, then increased to 75 °C with a 5°C/min heating rate and kept at this temperature for 2 min, and then increased to a final temperatureof 100 °C and kept at this temperature for 10 min.

Conversion (c) and enantiomeric excess of the substrate (ee _S_ ) were calculated as given in Eqs. (1) and (2), where C _S_ , C _R_ ,C _S0_ , and CR0represent the (S)-2-pentanol, (R)-2-pentanol, initial (S)-2-pentanol, and initial (R)-2-pentanol concentrations in mM, respectively.

(1)c%=(1-CS+CRCS0+CR0)x100

(2)ees%=CS-CRCS+CRx100

### 2.4. Mathematical method

The proposed mathematical model for the packed-bed bioreactor was built as a collection of simultaneous processes involving axial dispersion and nonlinear kinetic terms (Eq. (3)).The governing equation was written under the basic assumptions as isothermal and steady state conditions. The radial dispersion was negligible when compared with the axial dispersion [12,13]. It was assumed that the axial dispersion coefficientwas constant throughout the reactor, and the change in pellet size was omitted.

(3)Dzd2CRdz2-U0εdCRdz+r''meεV=0

In Eq. (3), D _z_ is the axial dispersion coefficient, C _R_ is the substrate concentration for (R)-2-pentanol, z is the axial coordinate, U _0_ is the superficial fluid velocity,ε is the void fraction of the packed-bed reactor, r″ is the apparent reaction rate per mass of the catalysts and time, m _e_ is the mass of enzyme, and V is the volume of the reactor. For this reaction, in accordance with results, the process was assumed as irreversible with no product inhibition [3]. It was also assumed that the effective diffusivity of the substrate species inside of the porous support was constant.

The boundary conditions used for the model solution were as follows: C _A_ , C _A0_ , and L represent the vinyl butyrate, initial vinyl butyrate concentration, and length of the reactor, respectively [14].

(4)z=0CR=CR0; orCA=CA0

(5)z=LdCRdz=0ordCAdz=0

In this study, D _z_ was estimated from a plot in which D _z_ ε/U _0_ d _p_ was plotted versus the Reynolds number [15].Here, d _p_ is the particle diameter (0.5 mm).

### 2.5. Reaction kinetics

The kinetic model used in this study was obtained from a previous study [3] and is represented in Eq. (6).

(6)r''=4.16CRCA51.17CR+103.73CA+CRCA

Here, r″ is the apparent reaction rate (mmole substrate/gr _enzyme_ /min).

The reaction kinetics were investigated in previous work about the transesterification of (R,S)-2-pentanol. A Ping Pong Bi Bi mechanism was found to be suitable for describing this reaction.

### 2.6. Productivity

Productivity (P) indicates the amount of product that can be formed by 1 g of immobilized enzyme per minute. It is one of the comparison methods of the continuous-flow and batch processes. Productivity was calculated at any degree of conversion. The same degree of conversions was used for both processes for the same concentration [2].

To compare the batch and continuous-flow processes of the transesterification of (R,S)-2-pentanol, productivities were calculated from Eqs. (7) and (8), where C _P_ , Q, n, and t represent the product ((R)-2-pentyl butyrate) concentration, volumetric flow rate, mole of the product ((R)-2-pentyl butyrate),and reaction time, respectively. For batch process in which the same immobilized enzyme was used, the values were taken from previous work [3].

(7)Pcontinuous=CPxQme

(8)Pbatch=ntxme

## 3. Results and discussion

In this study, the kinetic resolution of (R,S)-2-pentanol was performed fora continuous-flowpacked-bed reactor. Effects of the residence time, substrate concentration, and operation time of the enzyme were also investigated.

### 3.1. Residence time (flow rate) effect

**Figure 2:**
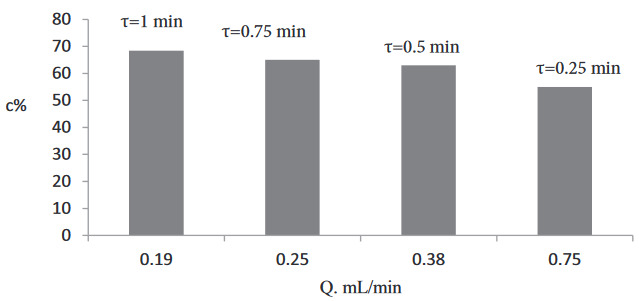
Effect of the residence time on conversion (200 mM (R,S)-2-pentanol, 200 mM vinyl butyrate, T = 30 °C, 270 mg Novozyme 435).

**Figure 3:**
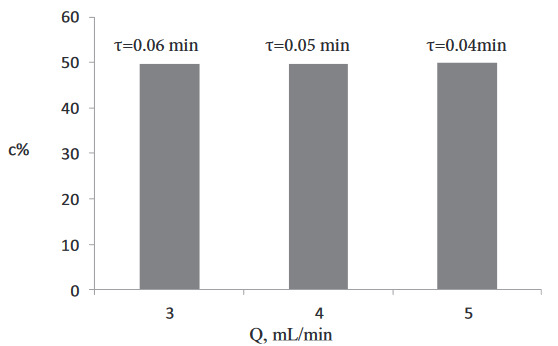
Effect of the flow rate on conversion (300 mM (R,S)-2-pentanol, 300 mM vinyl butyrate, T = 30 °C, 270 mg Novozyme 435).

To investigate the effect of the residence time on the conversion and enantioselectivity, first, kinetic resolution was performed at with an equimolar substrate concentration (200 mM of racemic-2-pentanol, 200 mM of vinyl butyrate)at various residence times (1, 0.75, 0.5, and 0.25 min). The conversion obtained washigher than 50% (Figure 2). Therefore, to achieve 50% conversion, further experiments were conducted at 3 different lower residence times (0.04, 0.05, and 0.06 min), and 50% conversion and>99% eeS were obtained at these lower residence times (Figure 3).

Thus, to investigate the substrate concentration effect on the kinetic resolution, experiments were conducted at a lower residence time (0.04 min, 5 mL/min).

### 3.2. Effect of substrate concentration

**Figure 4:**
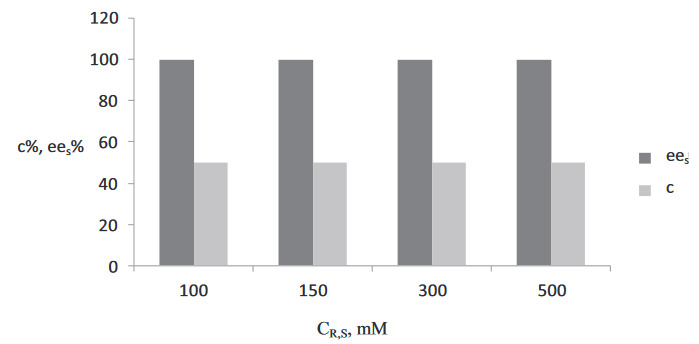
Effect of the substrate concentration ((R,S)-2-Pentanol) on the ee _S_ and conversion at τ = 0.04 min (T = 30 °C, Q = 5 mL/min, 270 mg Novozyme 435).

The effect of the substrate concentration was investigated with different concentrations of (R,S)-2-pentanol (C _R,S_ ) and vinyl butyrate as the acyl donor (C _A_ ) in equimolar mixtures (100, 150, 200, and 500 of mM in n-hexane) at a flow rate of 5 mL/min. Data on the conversion and ee _S_ for different substrate concentrations are presented in Figure 4 for the 4 substrate concentrations. As a result, 50% conversion and >99% ee _S_ were obtained at a residence time of 0.04 min (5 mL/min). In aprevious study, the eeS was obtained as approximately 99% in a batch system with a substrate concentration of 150 mM for 30 min (50% conversion) [3].

### 3.3. Productivity

Continuous-flow operation allows higher production capacity and better productivity than that of a batch reactor. It has beenreported that a continuous-flow packed-bed reactor offers higher selectivity and productivity [16,17]. Therefore, to compare the batch and continuous-flow processes, the experiments herein were conducted with a substrate concentration of 150 mM for both of the processes and the values at 50% conversion were used for the productivity calculations (Eqs. (7) and (8)). According to the results, productivity in the continuous-flow mode (1.341 mmol/min/g) was higher than that of the batch mode (0.363 mmol/min/g) by approximately 4 times (Table). It has been suggested that this was due to higher catalyst loading (g of catalyst per mL of reaction medium) in continuous-flow packed-bed reactors than in batch reactors. Ma et al. studied the production of enantiopure 2-hydroxyacids in a continuous-flow packed-bed reactor, and they also reported that high productivity was obtained in continuous-flow mode [11].

**Table:** Comparison of the batch and continuous-flow modes.

Continuous-flow mode	Batch mode
Q, mL/min	C _0_ , mM	P, mmol/min/g	C _0_ , mM	P, mmol/min/g
3	150	0.789	150	0.363
4	1.064
5	1.341

### 3.4. Reactor modeling

In this study, axial dispersion was used as the packed-bed reactor design equation. In the reactor models, a Ping Pong Bi Bi model was used as the kinetic term. Due to the short length of the reactor, samples were taken from upstream under steady state conditions. The void fraction ε) of the packed-bed reactor was measured as 0.3. m( _g_ is 270 mg of Novozyme 435, 0.5 mm of the particle diameter,and V 0.628 mL of the total volume. As mentioned in Section 2.4, the D _z_ and U _0_ ε values at a flow rate of 5 mL/min (τ=0.04 min) and Reynolds number of 7.69 (d/ _p_ U _0_ r/m) were estimated as 2.21× 10 ^–5^ m ^2^ /s and 0.022 m/s, respectively.

**Figure 5:**
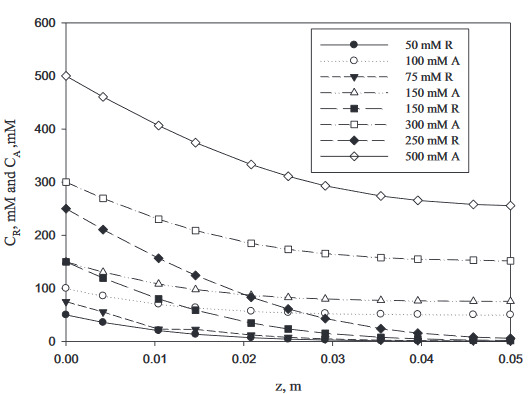
Variations of the concentration obtained from solving Eqs. (9) and (10) with the axial length of the packed-bed rector for a flow rate of 5 mL/min (T = 30 °C, τ = 0.04 min).

Experiments were conducted at a flow rate of 5 mL/min with 4 different substrate concentrations ((R)-2-pentanol at 50, 75, 150, and 250 mM; vinyl butyrate at100, 150, 300, and 500 mM) in a reactor with a L/D of 12.5.The mathematical models for the (R)-2-pentanol (R) and acyl donor (vinyl butyrate) (A) are given in Eqs. (9) and (10), respectively. These equations were solved using an algorithm for solving boundary value problems for ordinary differential equations by collocation (bvp4c) using MATLAB. The results are given in Figure 5. As the substrate conversion in the studied concentration range was 50%, the model and experimental data were in agreement. It was concluded that the model was suitable for the continuous-flow packed-bed reactor.

(9)Dzd2CRdz2-U0εdCRdz+4.16CRCAmg1000V∈(51.17CR+103.73CA+CRCA)60=0

(10)Dzd2CAdz2-U0εdCAdz+4.16CRCAmg1000V∈(51.17CR+103.73CA+CRCA)60=0

## 4. Conclusion

In this study, the kinetic resolution of 2-pentanol to achieve (S)-2-pentanol, which is a key intermediate required in the synthesis of several potential anti-Alzheimer drugs that inhibitβ-amyloid peptide release or synthesis, was investigated in a packed-bed reactor.

When compared to the batch reactor (0.363 mmol/min/g), higher productivity (1.341 mmol/min/g) was achieved in the continuous-flow reactor.The process parameters for the continuous-flow packed-bed reactor were also optimized to identify the best operating conditions.

Furthermore, the mathematical model of the continuous-flow packed-bed reactor was developed as an axial dispersion modeland it was solved using MATLAB. This model can be used for the industrial application of bioprocess engineering.
